# M-Current Suppression, Seizures and Lipid Metabolism: A Potential Link Between Neuronal Kv7 Channel Regulation and Dietary Therapies for Epilepsy

**DOI:** 10.3389/fphys.2020.00513

**Published:** 2020-05-25

**Authors:** Naoto Hoshi

**Affiliations:** ^1^Department of Pharmaceutical Sciences, University of California, Irvine, Irvine, CA, United States; ^2^Department of Physiology and Biophysics, University of California, Irvine, Irvine, CA, United States

**Keywords:** Kv7 channel, regulation – physiological, valproic acid, epilepsy, medium chain fatty, palmitoylation

## Abstract

Neuronal Kv7 channel generates a low voltage-activated potassium current known as the M-current. The M-current can be suppressed by various neurotransmitters that activate Gq-coupled receptors. Because the M-current stabilizes membrane potential at the resting membrane potential, its suppression transiently increase neuronal excitability. However, its physiological and pathological roles *in vivo* is not well understood to date. This review summarizes the molecular mechanism underlying M-current suppression, and why it remained elusive for many years. I also summarize how regulation of neuronal Kv7 channel contributes to anti-seizure action of valproic acid through inhibition of palmitoylation of a Kv7 channel binding protein, and discuss about a potential link with anti-seizure mechanisms of medium chain triglyceride ketogenic diet.

## Introduction

The M-current is a low voltage-activated potassium current generated by combination of neuronal KCNQ/Kv7 subunits (Kv7.2, 7.3, 7.4, and 7.5; Jentsch, 2000; Delmas and Brown, 2005; Greene and Hoshi, 2017). Since the activation threshold of neuronal Kv7 channels is near the resting membrane potential, activation of this channel strongly antagonizes membrane depolarization, and neuronal firing. Several neurotransmitters and hormones suppress neuronal Kv7 channel activity via Gq-coupled GPCRs, a phenomenon commonly known as M-current suppression, which diminishes its negative regulation, and creates temporal increase in neuronal firings (Delmas and Brown, 2005). Because M-current suppression causes drastic increase in neuronal excitability, M-current suppression has been considered to have a significant impact on central nervous functions. However, because there were no tools to prevent M-current suppression until very recently, physiological and pathological roles of M-current suppression *in vivo* are not well understood. Since this is a special issue for Kv7 channels covering various aspects, this review will focus on M-current suppression and its contribution to anti-seizure action of valproic acid.

## Molecular Mechanism of M-Current Suppression

Despite its clear physiological effects on neuronal excitability, elucidating molecular mechanism underlying M-current suppression took many years. The first proposed candidate was a protein kinase C (PKC)-mediated mechanism, where activation of PKC by phorbol esters can suppress M-current (Higashida and Brown, 1986). It seemed to be a plausible mechanism because Gq-coupled receptors activate phospholipase C (PLC), which then activates PKC. However, follow up studies from several labs showed that PKC inhibitors do not disrupt M-current suppression, which undermined this mechanism (Bosma and Hille, 1989; Shapiro et al., 2000; Stemkowski et al., 2002).

A next proposed mechanism was by intracellular calcium. Treatments that rise intracellular calcium has been shown to suppress the M-current (Yu et al., 1994; Selyanko and Brown, 1996; Cruzblanca et al., 1998). In addition, calmodulin, a calcium sensing protein, was identified as an auxiliary subunit for Kv7 channels (Wen and Levitan, 2002; Yus-Najera et al., 2002). Furthermore, co-expression of the calcium insensitive mutant calmodulin diminished calcium-induced M-current suppression (Gamper and Shapiro, 2003). These studies clearly demonstrate that intracellular calcium can suppress the M-current and mediates M-current suppression induced by some receptors. However, inhibiting calcium responses failed to disrupt M-current suppression induced by m1 muscarinic receptors (Shapiro et al., 2000) or by purinergic ATP receptor (Stemkowski et al., 2002), which suggests that this is not the universal mechanism.

Next candidate mechanism involves phosphatydilinositol-4,5-bisphosphate (PIP2). Phosphoinositides are minor acidic phospholipids with an inositol sugar head (Falkenburger et al., 2010). Depending on the positions and numbers of phosphorylation of the inositol sugar head, phosphoionsitides show distinct physiological functions on various biological processes ranging from endocytosis, cell growth, to membrane protein regulation at various membrane compartments (Suh and Hille, 2008). PIP2 is the lipid substrate for PLC, but it is also an essential co-factor for various transporters and ion channels (Falkenburger et al., 2010). Kv7 channel family is one example of these proteins, and cannot transduce potassium ion without PIP2 binding (Suh and Hille, 2008; Sun and MacKinnon, 2020). Since PIP2 is consumed by PLC upon Gq-coupled receptor activation, depletion of PIP2 can generate M-current suppression (Suh and Hille, 2002; Zhang et al., 2003). In addition, enzymatic increase in PIP2 concentration at the plasma membrane can partially disrupt M-current suppression, which supports the PIP2 depletion hypothesis (Suh et al., 2006). However, this also raises a question: if PIP2 is an essential co-factor for many ion channels and transporters, how can PIP2 regulate M-current without having off-target side effects?

**FIGURE 1:**
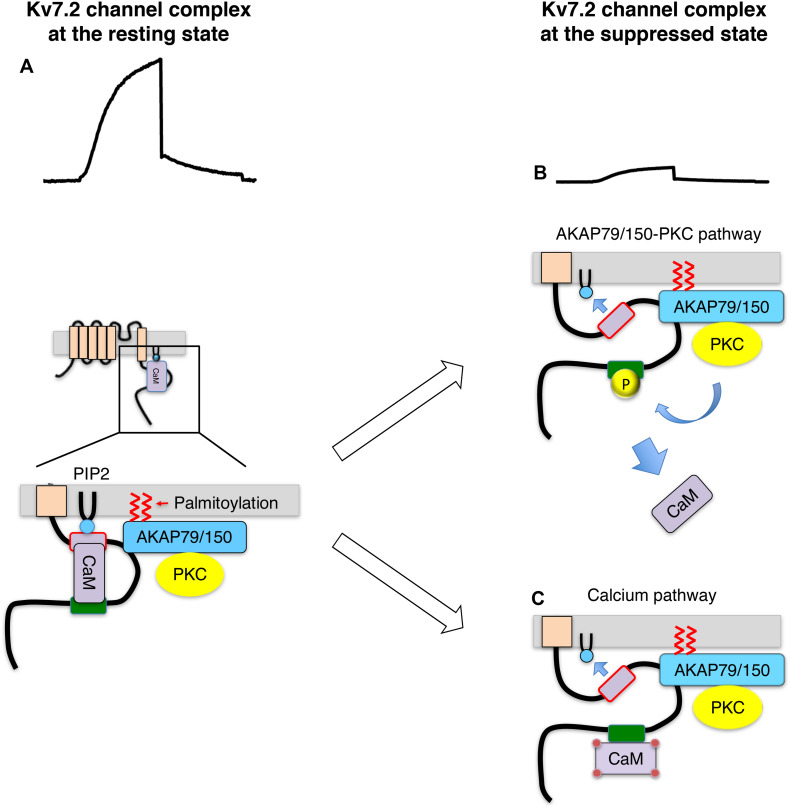
Summary of Kv7.2 channel protein complex and its changes during M-current suppression, modified from ref (Kosenko et al., 2012). **(A)** Kv7.2 channel complex at the resting state. **(B)** Suppressed Kv7.2 channel through AKAP79/150-PKC pathway. Kv7.2 subunit is phosphorylated by AKAP79/150 anchored PKC when Gq-coupled receptors are activated. Phosphorylated Kv7.2 subunits release CaM, which reduces PIP2 binding to Kv7.2 subunit reducing functioning channels. **(C)** Suppressed Kv7.2 channel through intracellular calcium. Some Gq-coupled receptors suppress M-current via increase in intracellular calcium. Calcium-bound CaM changes configuration of Kv7.2 binding, which reduces PIP2 binding to Kv7.2 subunit.

Soon after this discovery, a related but distinct pathway was found involving a scaffold protein, AKAP79/150 (79 human/150 rodent). AKAP79/150 is a scaffold protein that anchors protein kinase A (PKA), PKC, protein phosphatase 2B (PP2B/calcineurin) and calmodulin, and has identified as a Kv7.2 binding protein (Hoshi et al., 2003). AKAP79/150 locates at the inner surface of the plasma membrane through PIP2 binding and attached fatty acid by protein palmitoylation (Dell’Acqua et al., 1998; Wong and Scott, 2004; Keith et al., 2012). It has been shown that activation of muscarinic acetylcholine receptor requires Kv7.2 channel anchored PKC via AKAP79/150 for M-current suppression (Hoshi et al., 2003; Hoshi et al., 2005; Kosenko et al., 2012). The identified pathway is summarized as follows (Figures 1A,B; Kosenko et al., 2012): activation of Gq-coupled receptor activates AKAP79/150 anchored PKC and phosphorylates Kv7.2 subunits. PKC phosphorylates target serine residues of Kv7.2 subunits including one located at the calmodulin binding site. Phosphorylation of the conserved serine residue at the calmodulin binding site interferes with calmodulin binding, which leads to a change in CaM-Kv7 configuration that reduces the affinity of Kv7.2 subunit toward PIP2. Thus, Kv7 channel activity is suppressed. This Kv7.2-AKAP79/150-calmodulin complex also provides a molecular mechanism illustrating calcium-induced M-current suppression. Namely, calcium-bound calmodulin changes CaM-Kv7.2 configuration and reduces affinity of Kv7.2 to PIP2 (Figure 1C; Kosenko and Hoshi, 2013). Together, Kv7.2 channel complex integrates distinct signals to decrease in sensitivity of Kv7 channel toward PIP2, which leads to M-current suppression. This mechanism explains how a ubiquitous cofactor, PIP2, can selectively regulates Kv7 channels.

The Kv7.2-AKAP79/150-PKC protein complex not only provided insights as to how distinct stimuli suppress the M-current, but it also explains why PKC inhibitors could not disrupt M-current suppression in past studies. It revealed that AKAP79/150 binding to PKC protects PKC molecules from commonly used PKC inhibitors (Hoshi et al., 2010). In summary, PIP2 is an important regulator of Kv7 channel activity. Reduction of PIP2 level or sensitivity of Kv7 channel to PIP2 can reversibly induce M-current suppression.

## Inhibition of S-Palmitoylation of AKAP79/150 Contributes to Anti-Seizure Effects of Valproic Acid Through Kv7.2 Channels

**FIGURE 2:**
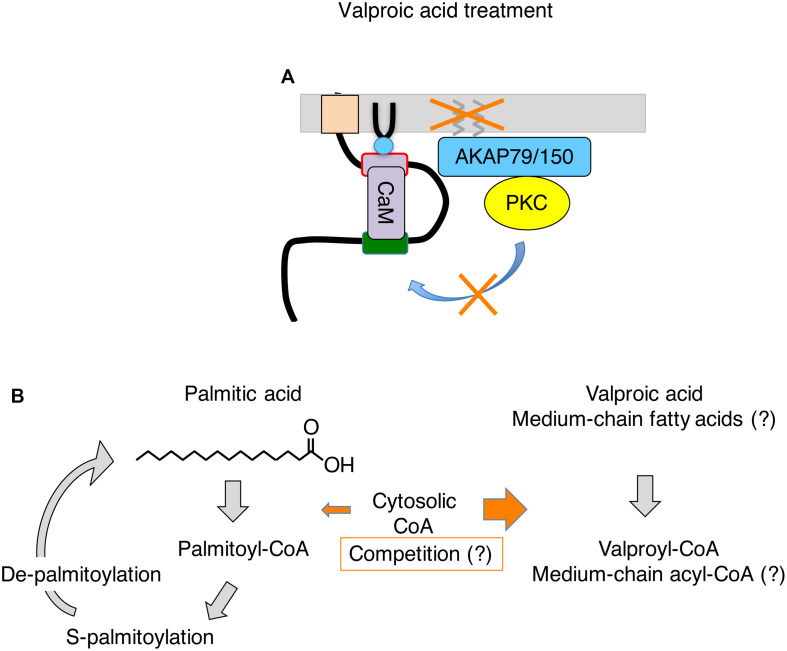
Summary of events induced by valproic acid treatment on Kv7.2 channel complex. **(A)** Valproic acid treatment inhibits S-palmitoylation of AKAP79/150. Palmitoylation-deficient AKAP79/150 cannot mediate PKC phosphorylation of Kv7.2 subunit, which preserves M-current during seizures. **(B)** Summary of S-palmitoylation cycle and a potential action of valproic acid in this process. Valproic acid treatment may deplete free cytosolic CoA and interfere with generation of palmitoyl-CoA. A similar mechanism may be applicable to MCT therapy. See text for detail.

Valproic acid is a branched short chain fatty acid, and is one of the most commonly prescribed anti-epileptic drugs used for many decades (Perucca, 2002). Various mechanisms have been proposed to explain its anti-seizure effects such as enhancement of inactivation of sodium channel, increase in GABA content in the brain, changing fatty acid metabolism, and inhibition of HDAC (Perucca, 2002; Silva et al., 2008). However, the exact mechanism of action is not well understood to date. Recently, multi-day treatment with valproic acid has been shown to disrupt M-current suppression, which contributes to its anti-seizure action *in vivo* (Kay et al., 2015). In this mechanism, valproic acid inhibits a subset of protein palmitoylation in the brain including AKAP79/150. Because palmitoylation of AKAPa79/150 is required for phosphorylation of Kv7.2 subunit by AKAP79/150-anchored PKC, deficient palmitoylation preserves the M-current from suppressive neurotransmitters during seizures, which prevents aggravation of seizures (Kay et al., 2015; Figure 2A). A follow-up study using Kv7.2 mutant knock-in mice that lack the target PKC acceptor serine, Kv7.2(S559A), showed no further anti-seizure effect of valproic acid treatment (Greene et al., 2018), which confirmed that valproic acid acts on the AKAP79/150-PKC-Kv7.2 pathway. This study also suggests that neuronal Kv7 channel is the major effector for non-acute anti-seizure action of valproic acid treatment.

## Regulation of AKAP79/150 Palmitoylation and Fat Metabolism in Neuron

S-Palmitoylation is a posttranslational modification that attaches long-chain fatty acids (C14,16, and 18), predominantly palmitic acid (C16), to cysteine residues through a thioester bond (Linder and Deschenes, 2007). Palmitoylation of proteins facilitates membrane localization at various membrane compartments. For the plasma membrane, protein palmitoylation facilitates its localization at the lipid raft, signaling hot spot (Linder and Deschenes, 2007). S-palmitoylation is characterized by cycles of palmitoylation and de-palmitoylation of the same protein molecules in response to cellular stimuli, which distinct itself from other one-time lipid modifications such as myristoylation (Linder and Deschenes, 2007). S-palmitoylation is mediated by DHHC palmitoyl acyltransferases utilizing long fatty acid-CoA as substrates (Fukata and Fukata, 2010). Among 23 members of DHHC enzymes, DHHC2 has been shown to mediate palmitoylation of AKPA79/150 (Woolfrey et al., 2015). Palmitoylation of AKAP79/150 at dendrites in neurons are regulated by neuronal activities, which correlates well with glutaminergic synaptic strength (Keith et al., 2012). Stimuli that cause long-term potentiation of synapses increase palmitoylation of AKAP79/150, while stimuli that cause long-term depression of synapses enhance de-palmitoylation. These findings suggest that palmitoylation of AKAP79/150 is in a dynamic equilibrium, which requires constant supply of palmitoyl-CoA.

Despite the fact that 60% of brain weight is lipid, neurons have a surprisingly low capacity for lipid metabolism including beta-oxidation in mitochondria or lipid synthesis (Belanger et al., 2011; Schonfeld and Reiser, 2013; Bruce et al., 2017; Romano et al., 2017). In addition, acyl-CoA in neurons is maintain at very low concentration, otherwise leads to neurodegeneration (Ellis et al., 2013). This low capacity of processing lipids in neurons implies that substrates for lipid processes including membrane synthesis or palmitoylation must be supplied directly and promptly from the cerebrospinal fluid or surrounding astrocytes rather than synthesized inside the neurons (Belanger et al., 2011). Increasing evidence suggests that neuron-astrocyte metabolic coupling plays an important role for supplying fatty acids, and removing excess lipid from neurons (Belanger et al., 2011; Ioannou et al., 2019). Therefore, if valproic acid changes substrate supplies such as palmitic acid, it would have drastic effects on neurons, which suggests that the site of action for valproic acid can be metabolically active non-neuronal cells such as astrocytes.

## Crossroad for S-Palmitoylation, Valproic Acid and Medium Chain Fatty Acids

Valproic acid has various pharmacological effects, as mentioned earlier, and conditions that complicate pinning down its mechanism of action. For instance, the optimal therapeutic serum concentration of valproic acid is 300 to 700 μM (Perucca, 2002), which is significantly higher than other therapeutic compounds that function as ligands at a sub-micromolar range. Even though a concentration of valproic acid in the cerebrospinal fluid is 10 ∼ 30% of those in the serum (Loscher and Nau, 1983), valproic acid is most likely not a high-affinity ligand for target proteins. In addition, there are several processes where valproic acid can interfere with palmitoylation without acting as an enzyme inhibitor.

One potential mechanism is interference with coenzyme A (CoA) conjugation with long-chain fatty acids in the cytoplasm. Because valproic acid is a fatty acid, it is conjugated to CoA for processing. It is known that the cytosolic CoA is used for lipid synthesis, membrane trafficking and protein modification, and its concentration in mammalian cells is between 20–140 μM, while mitochondrial CoA, which is involved in energy production, is 2–5 mM (Leonardi et al., 2005). It has been shown that valproic acid can deplete free CoA and interfere with lipid metabolism including beta oxidation (Silva et al., 2008). In addition, *in vitro* study shows that valproic acid inhibits long fatty acid acyl transferase in brain microsomes by reducing palmitoyl-CoA (Bazinet et al., 2006). Therefore, high occupancy of CoA by valproic acid may interfere with supply of palmitoyl-CoA.

On the other hand, decreasing the palmitoyl-CoA pool in neurons may be achieved by conditions unrelated to valproic acid. Similar scenarios discussed above may be applied to shorter fatty acids that are not suitable as a substrate for palmitoylation. Dietary therapies known as ketogenic diet have been used to treat drug refractory epilepsy. These diets are composed of a high fat diet, reduced carbohydrate intake, and induction of ketosis (Warren et al., 2018). A modern improvement of ketogenic diet was addition of medium chain fatty triglycerides (MCT) in the therapy, which are rich in octanoic acid (C8), and decanoic acid (C10). MCT was originally added to the regimen because they are more ketogenic than long chain triglycerides (Huttenlocher et al., 1971). However, it has been shown that both octanoic acid (Wlaz et al., 2012), and decanoic acid (Chang et al., 2016) have anti-seizure effects without inducing ketosis. In addition, it has been shown that MCT diet can raise serum concentration of octanoic acid to 300 μM and decanoic acid to 100 μM (Haidukewych et al., 1982), which is above concentration of cytosolic CoA and similar to the therapeutic concentration of valproic acid. Furthermore, triheptanoin, a triglyceride contains three heptanoates (C7), also has anti-seizure effects (Borges and Sonnewald, 2012), which generates three heptanoyl-CoA per compound. These pieces of emerging evidence suggest that medium chain fatty acids themselves are anticonvulsants. Various hypotheses are proposed as a mechanism for anti-seizure effects of these medium chain fatty acids ranging from changing energy production, neurotransmitter balance and ion channel activities (Warren et al., 2018). However, I would like to point out that carbon numbers of these anticonvulsant medium chain fatty acids (C7-C10) are outside of the acceptable carbon length of S-acyltransferase (C12-C18) that mediate protein palmitoylation (Rana et al., 2018). If these medium chain fatty acids can dominate cytosolic acyl-CoA pool and reduce palmitoyl-CoA, it would suppress palmitoylation of AKAP79/150 and have a similar outcome achieved by valproic acid. Only difference would be that medium chain fatty acids can be metabolized in various cells other than hepatocytes.

Based on the discussion above, the purpose of the ketogenic diet therapy for anti-epileptic treatment may not be creating a ketosis, but may be producing shorter fatty acids, which changes palmitoylation profiles in neurons. In summary, identification of palmitoylation as a contribution factor for anti-seizure action of valproic acid provides a prototypical mechanism for linking lipid metabolism and anti-seizure action. Further studies are required to determine whether MCT ketogenic therapy induce similar changes in palmitoylation profile of neuronal proteins.

## Author Contributions

The author confirms being the sole contributor of this work and has approved it for publication.

## Conflict of Interest

The author declares that the research was conducted in the absence of any commercial or financial relationships that could be construed as a potential conflict of interest.
